# Accuracy of registration techniques and vascular imaging modalities in fusion imaging for aortic endovascular interventions: a phantom study

**DOI:** 10.1186/s42155-021-00234-6

**Published:** 2021-06-14

**Authors:** M. M. Sieren, C. Schareck, M. Kaschwich, M. Horn, F. Matysiak, E. Stahlberg, F. Wegner, T. H. Oechtering, J. Barkhausen, J. Goltz

**Affiliations:** 1 Department for Radiology and Nuclear Medicine, University Hospital of Schleswig-Holstein, Campus Lübeck, Ratzeburger Allee 160, 23562 Lübeck, Germany; 2 Department for Vascular Surgery, University Hospital of Schleswig-Holstein, Campus Lübeck, Lübeck, Germany; 3 Department for Radiology and Neuroradiology, Sana Hospital, Lübeck, Germany

**Keywords:** Fusion imaging, Anthropomorphic body phantom, Registration accuracy, 2D-3D registration, 3D-3D registration

## Abstract

**Background:**

This study aimed to assess the error of different registration techniques and imaging modalities for fusion imaging of the aorta in a standardized setting using a anthropomorphic body phantom.

**Materials and methods:**

A phantom with the 3D printed vasculature of a patient suffering from an infrarenal aortic aneurysm was constructed. Pulsatile flow was generated via an external pump. CTA/MRA of the phantom was performed, and a virtual 3D vascular model was computed. Subsequently, fusion imaging was performed employing 3D-3D and 2D-3D registration techniques. Accuracy of the registration was evaluated from 7 right/left anterior oblique c-arm angulations using the agreement of centerlines and landmarks between the phantom vessels and the virtual 3D virtual vascular model. Differences between imaging modalities were assessed in a head-to-head comparison based on centerline deviation. Statistics included the comparison of means ± standard deviations, student’s t-test, Bland-Altman analysis, and intraclass correlation coefficient for intra- and inter-reader analysis.

**Results:**

3D-3D registration was superior to 2D-3D registration, with the highest mean centerline deviation being 1.67 ± 0.24 mm compared to 4.47 ± 0.92 mm. The highest absolute deviation was 3.25 mm for 3D-3D and 6.25 mm for 2D-3D registration. Differences for all angulations between registration techniques reached statistical significance. A decrease in registration accuracy was observed for c-arm angulations beyond 30° right anterior oblique/left anterior oblique. All landmarks (100%) were correctly positioned using 3D-3D registration compared to 81% using 2D-3D registration. Differences in accuracy between CT and MRI were acceptably small. Intra- and inter-reader reliability was excellent.

**Conclusion:**

In the realm of registration techniques, the 3D-3D method proved more accurate than did the 2D-3D method. Based on our data, the use of 2D-3D registration for interventions with high registration quality requirements (e.g., fenestrated aortic repair procedures) cannot be fully recommended. Regarding imaging modalities, CTA and MRA can be used equivalently.

## Introduction

Endovascular therapy for vascular pathologies has become an established technique on par with surgical therapy in many vascular territories (Feezor et al. 2007; Indes et al. 2013; Mandawat et al. 2012). While the minimally invasive approach carries advantages for the patient, contrast media (CM) poses a health risk to the patient and ionizing radiation for both the patient and the interventionist (Brooks et al. 2011; Gleeson and Bulugahapitiya 2004; Kawatani et al. 2016; Solomon and Dumouchel 2006). Fusion imaging (FI) is becoming more established as an add-on technique for significantly reducing CM doses and radiation exposure in various endovascular procedures (Stahlberg et al. 2019; Goudeketting et al. 2018; Sailer et al. 2015; Swerdlow et al. 2019; Goudeketting et al. 2017). Nonetheless, significant inaccuracies in fusion overlays have been reported (Schulz et al. 2016).

One of the main challenges of FI is sufficient registration of fluoroscopic or cone-beam computed tomography images with pre-interventionally acquired cross-sectional imaging (Sailer et al. 2014; Abi-Jaoudeh et al. 2012). The two established registration methods (2-dimensional-3-dimensional (2D-3D) registration; 3D-3D registration) differ in the resulting accuracy, but also in the technical complexity of the registration and the required radiation doses. (Goudeketting et al. 2017). Although individual accuracy measurements have been performed in sub-analyses of some studies (Schulz et al. 2016; Schwein et al. 2018; Tacher et al. 2013), most studies have focused on the reduction of CM and radiation dose. Literature that systematically evaluates the accuracy of image registration techniques is scarce (Schulz et al. 2019). Moreover, pre-interventionally acquired CTA and MRA data are employed for FI; possible differences between both modalities have not been systematically addressed to date.

Therefore, this study aimed to compare 2D-3D and 3D-3D registration techniques using CTA and MRA data in a standardized setting in an anthropomorphic body-vascular phantom to evaluate FI’s quality in the thoracic and the abdominal vasculature.

## Materials and methods

### Phantom

**Fig. 1:**
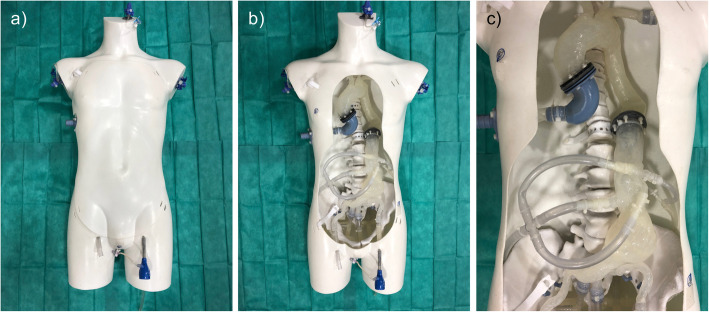
Depiction of the true-to-life body phantom: **a** with cover; **b** without the cover; and **c** a close-up of the vasculature. The phantom was derived from a CT angiography dataset of a female patient suffering from an infrarenal aortic aneurysm. The skeleton was manufactured using 3D printing that was equipped with radiopaque coating. Vessel entry was possible via removable sluices in the communal femoral arteries, the ascending aorta and the supra-aortic vessels

The phantom was composed of a radiopaque skeleton and a 3D vascular model (Fig. 1). The vascular model was created from a CTA dataset of a patient with an infrarenal abdominal aortic aneurysm (female, 174 cm, 72 kg, 82 years). The patient agreed to the use of her data via written informed consent. The phantom was produced by hybrid additive manufacturing based on fused deposition modeling (Felix 3, FELIXprinters), whereby the inner contours of the vessels were printed with a water-soluble material and then the model was covered with silicone (Shore A 37). The vessels represented include the aorta, supra-aortic, visceral, and iliac vessels. Vascular access was possible via the ascending aorta, the supra-aortic, and both external iliac vessels. The Phantom was connected to a diaphragm dosing pump, simulating pulsatile flow (Sigma, ProMinent®DeutschlandGmbH, Heidelberg). Breathing motion was not simulated.

### Computed tomography angiography

The CTA was performed on a 128-slice CT scanner (Somatom Definition AS+®, Siemens Healthcare, Erlangen). Imaging parameters were as follows: tube voltage = 120 kV; reference tube current-time product = 200mAs; rotation time = 0.3 s; collimation = 0.6 mm. The phantom was placed in the supine position and filled with an iodine CM (mixing ratio CM/0.9% sodium chloride 1/15; Imeron 300®, Bracco). Images were reconstructed employing a soft tissue kernel (B30f) and an effective slice thickness of 1.0 mm.

### Magnetic resonance angiography

A standard cartesian 3D fast field echo MR angiography was performed using a 3 T scanner (Philips Ingenia Omega dStream, Philips, Best) with a 20-channel body surface coil. Data were acquired in the axial plane for the thoracic and the abdominal regions. Acquisition parameters were as follows: field of view = 380x462x200mm; slice thickness = 5 mm; image matrix = 292 × 330; and time to repeat/time to echo = 3.8/2.4 ms. An acquired voxel size of 1.3 × 1.4 × 2.4 mm was reconstructed to 0.6 × 0.6 × 1.2 mm. The phantom contained blood mimicking fluid, consisting of 36.6% glycerine in 0.9% sodium chloride solution doped with CM (Gadubotrol, Gadovist®, Bayer, Leverkusen).

### Fluoroscopy and fusion image processing

Images were imported to a workstation in the angiography suite (Allura Xpert® FD20/15, 3.4, Phillips, Best). The following two steps had to be performed with dedicated software (VesselNavigator®, Phillips).

#### Planning

**Fig. 2:**
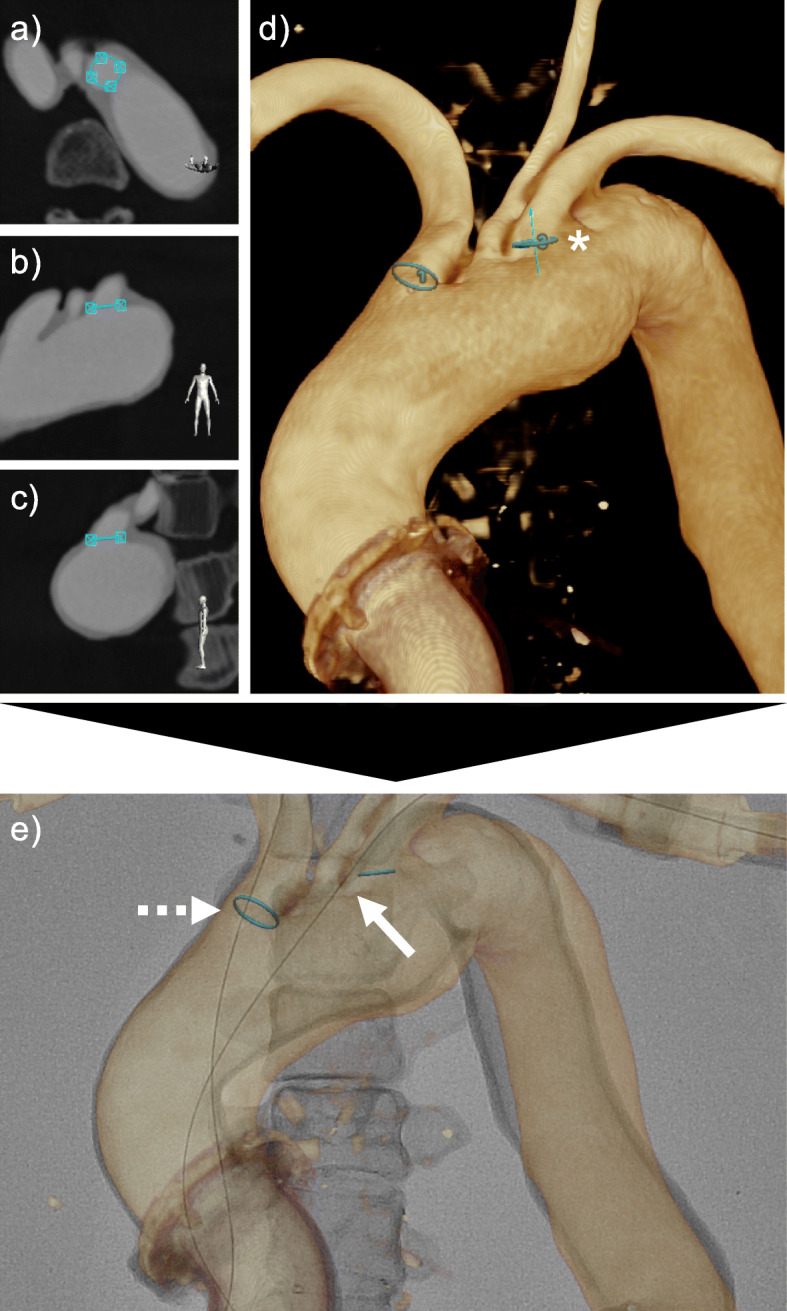
Exemplary placement of circular markers at vessel orifices and accuracy assessment via cannulation of those orifices with a guidewire. A circular marker was carefully adjusted to the anatomy of the left subclavian artery’s orifice using multiplanar reconstructions of the cross-sectional imaging data, in this case, a CTA (**a**–**c**). Each plane’s orientation is demonstrated by the human model in the bottom right corner of each image. **d** Correct placement of the marker (*) was verified in the vessel’s virtual 3D model, which was reconstructed from the original CTA data. For demonstration purposes, another marker was placed in the brachiocephalic trunk. **e** Subsequently, image fusion was performed, and each orifice was cannulated with a soft guidewire. If the wire went through the circular marker (brachiocephalic trunk; dashed arrow), the marker position was marked as accurate, and if the wire missed the marker (left subclavian artery; straight arrow), the position was dismissed as inaccurate

Vessel segmentation from CTA/MRA data was conducted semi-automatically and corrected manually, if necessary. The orifices of the vessel ramifications were marked via circular landmarks. Ideal placement of such a marker is shown in Fig. 2.

#### Registration

2D-3D fusion was performed by employing two fluoroscopic images in 90° right anterior oblique (RAO) and anterior-posterior projection. The 3D vessel model was consecutively fitted to fluoroscopic images using osseous landmarks.

Before 3D-3D fusion, a cone-beam CT was acquired in the angio-suite. Image fusion was achieved by correlating vessel-specific landmarks in both CT datasets (cone-beam CT; pre-interventional CT) as follows: In the thorax, ramifications of the supra-aortic vessels were used. In the abdomen, orifices of the visceral arteries were employed for alignment.

To test for inter-reader reproducibility, image fusion with each technique and image modality was performed by two investigators, blinded to the results of the other investigator. For the intra-reader analysis, the measurements were repeated by one investigator at an interval of 6 weeks.

### Data analysis

**Fig. 3:**
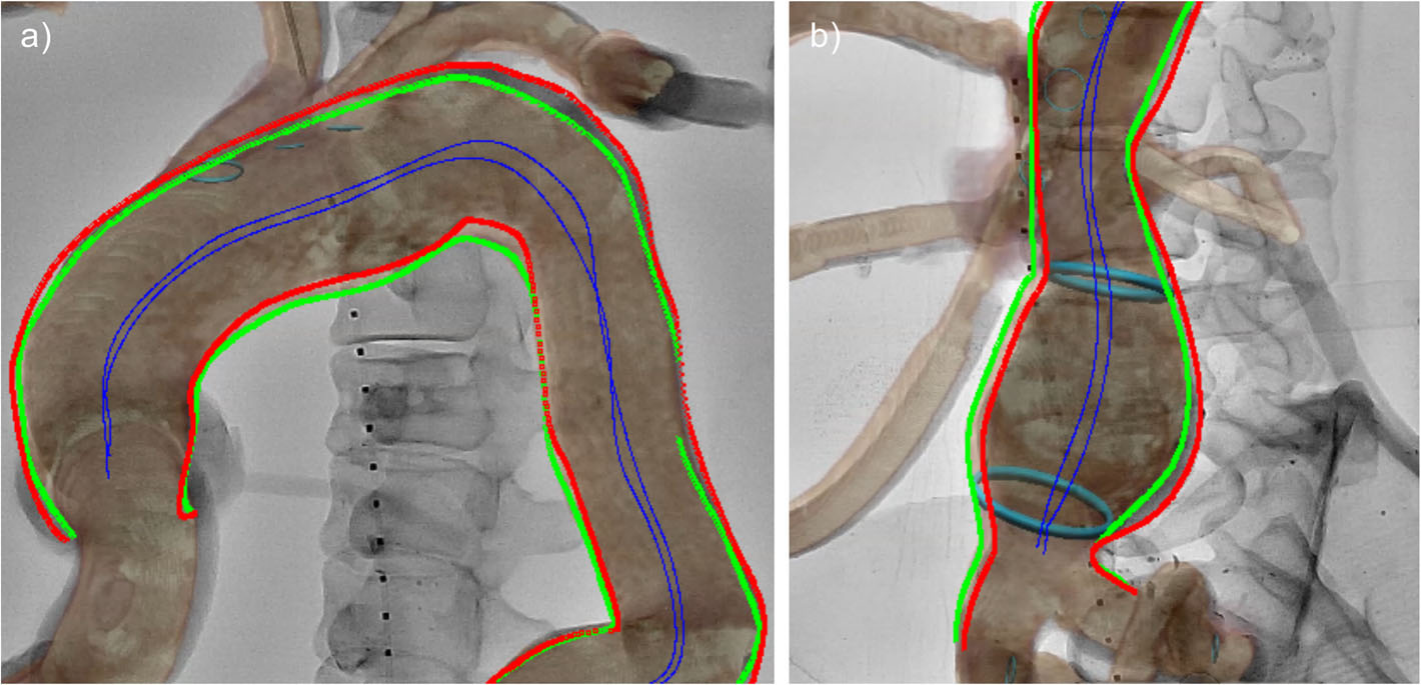
Analysis of centerline deviations between the actual vessel in the phantom and the virtual 3D model, as generated from an MRA after performing FI using the 2D-3D technique in **a** the thorax and **b** the abdomen. The actual vessel’s borders in the fluoroscopy image (red) and the virtual model (green) were outlined manually. A MatLab tool was used to generate a centerline for each vessel and to compare deviations between both centerlines (blue) on a pixel-wise basis every 10 mm. The analysis was repeated for seven angulations of the c-arm (− 90°, − 60°, − 30°, 0°, 30°, 60°, and 90°) in the thorax and the abdomen, respectively

To evaluate fusion quality, fluoroscopy of a full c-arm rotation from RAO to left anterior oblique (LAO; 90° to − 90°) was recorded at two levels of the phantom (thorax and abdomen). A custom MATLAB tool was used, which was validated using centerlines defined manually by three experts, based on the methodology presented here (Schaap et al. 2009). The mean error of the tool for centerline definition was 0.56 ± 0.34 mm. The tool calculated two centerlines after manually outlining the actual vessel’s borders and the virtual 3D vessel model on fluoroscopy images (Fig. 3). Deviation of these two centerlines was measured on a pixel-wise basis every 10 mm for seven c-arm angulations (− 90°, − 60°, − 30°, 0°, 30°, 60°, and 90°). The centerline defined on the fluoroscopy images served as reference standard for all accuracy measurements.

Accuracy of the landmark placement was evaluated on a binominal basis, as shown previously (Schwein et al. 2018; Chinnadurai et al. 2016). Landmarks were placed at each ramification of the aorta. A soft guidewire (GLIDEWIRE®, Terumo) was placed in the adjacent vessel, and a score of 0–1 was given, depending on the position of the wire (either in- or outside the circular marker, Fig. 2e).

### Statistics

Statistical analyses were performed using SPSS® (version 25.0, IBM Corp.).

Deviations of centerlines are presented as mean ± standard deviation. Differences between registration techniques and imaging modalities were tested for significance using student’s t-test. Significance was accepted at a *p*-value of < 0.05.

Graphs were calculated to illustrate the deviation of the centerlines for all c-arm angulations over the vessel’s course against the reference standard. Bland–Altman analysis, including calculation of mean bias and limits of agreement (mean bias±1.96*standard deviation), was performed to assess the differences between imaging modalities in head-to-head comparison.

To test for inter- and intra-reader reproducibility, the intraclass correlation coefficient (ICC) with 95% confidence intervals was calculated.

## Results

### Comparison of 2D-3D and 3D-3D registration

Detailed results of the deviations between centerlines can be found in Table 1.

**Table 1:**
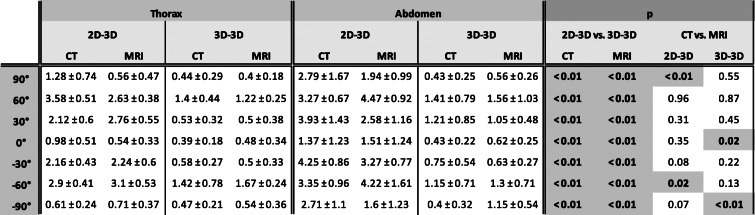
Comparison of different registration techniques and imaging modalities

Results for seven different c-arm angulations and both imaging modalities in the thorax and the abdomen are given. For each angulation, the mean ± standard deviation of all measurements along the vessel’s course was calculated. Significance was accepted for *p* < 0.05; significant differences are underscored in dark grey. Overall, 3D-3D registration was more accurate than 2D-3D registration. Furthermore, a decrease in registration accuracy was observed for c-arm angulations of 30° to 60° and − 30° to − 60°

**Fig. 4:**
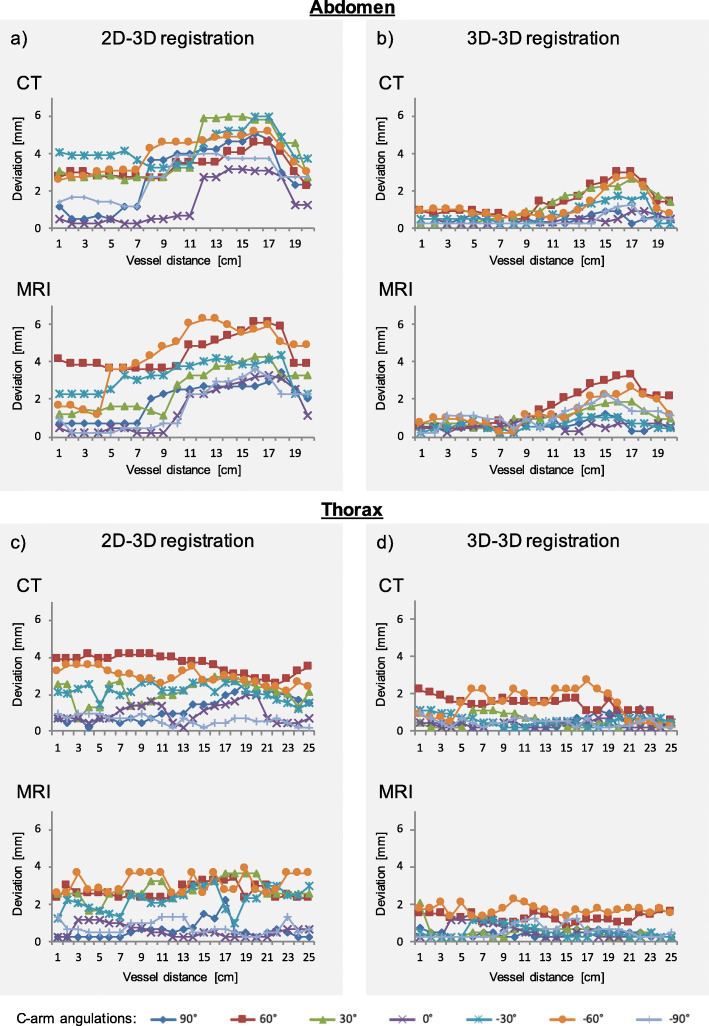
Graphical illustration of the centerline deviation of the virtual 3D model compared to fluoroscopy images of the thoracic (**a**, **b**) and abdominal (**c**, **d**) aortas. For each anatomical region, centerline deviations along the vessel course are given for both registration techniques (2D-3D; 3D-3D) and imaging modalities (CT; MRI), respectively. Each colored graph represents a c-arm angulation. The deviations between the centerlines were assessed along the vessel’s course every 10 mm. The measurements were performed in the thoracic aorta (from the aortic bulb to the diaphragm) and in the abdominal aorta (from the diaphragm to the aortic bifurcation)

The highest mean deviation of the centerlines for the 2D-3D technique was found for a c-arm angulation of − 60° for CT with 4.25 ± 0.86 mm and for an angulation of 60° for MRI with 4.47 ± 0.92 mm. The highest absolute deviation was 6.25 mm (MRI) and 5.95 mm (CT), recorded for a c-arm angulation of 60° in the abdomen. The highest mean deviations for 3D-3D registration were measured for CT with 1.42 ± 0.78 mm, and for MRI with 1.67 ± 0.24 mm. The highest absolute deviation was 3.25 mm (MRI) and 2.96 mm (CT), all recorded for a c-arm angulation of − 60° in the abdomen. Figure 4 illustrates the centerline deviations at every measurement point along the course of the aorta. All differences between registration techniques reached statistical significance.

A total of 32 (16 CT; 16 MRI) landmarks were evaluated for both registration techniques. In the 2D-3D registration, 26 landmarks (81%) were marked as accurate. The missed landmarks included the renal arteries, the superior mesenteric artery, and the left common carotid artery. In contrast, 32 landmarks (100%) achieved an accurate rating (score = 1) with 3D-3D registration.

### Comparison of CT and MRI

**Fig. 5:**
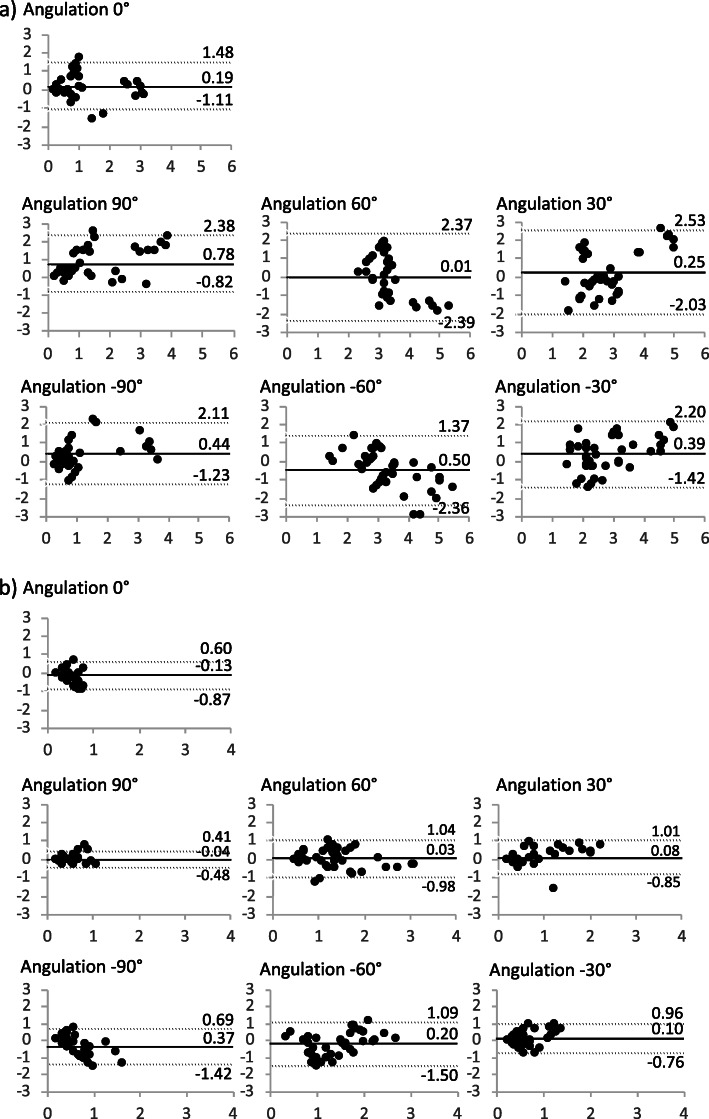
A Bland–Altman analysis of differences between imaging modalities for FI. For each c-arm angulation, differences between the centerline deviation between FI using the CTA and the MRA data for **a** 2D-3D registration and **b** 3D-3D registration were assessed. Means (straight line) and limits of agreement (mean ± 1.96*standard deviation; dashed line) are given. Overall, the spread of values was acceptably small, with the exception of individual values showing differences of > 3 mm using 2D-3D registration. 3D-3D registration was superior to 2D-3D registration regarding both the spread of values and the quality of agreement

Differences between means and standard deviations for the two different imaging modalities for both registration techniques were small (Table 1). Bias and limits of agreement in the Bland–Altman analysis can be found in Fig. 5. Although bias was below 1 mm for both registration techniques regarding the comparison of imaging modalities, values for the 3D-3D registration showed a lower spread and higher accuracy than 2D-3D registration. Significant differences between imaging modalities were observed for c-arm angulations of 90° and − 60° using 2D-3D registration, and of 0° and − 90° using 3D-3D registration.

There was no difference in the accuracy of landmark placement between both modalities.

### Inter- and intra-reader comparison

The ICC yielded excellent reliability of 0.89 (0.86–0.92) for inter-reader comparison between 2D-3D and 3D-3D registration and of 0.94 (0.89–0.99) between imaging modalities. ICC for intra-reader comparison was 0.96 (0.91–0.99).

## Discussion

This study demonstrates the quality of agreement of different registration techniques that are used for fusion imaging. For the first time, the present study confirms the equivalence of both CT and MRI data for fusion imaging in a standardized head-to-head comparison.

While the positive effects of fusion technology on radiation and contrast exposure are well established (Stahlberg et al. 2019; Goudeketting et al. 2018; Sailer et al. 2015; Swerdlow et al. 2019; Goudeketting et al. 2017), the present study focuses on assessing differences between the different registration techniques and imaging modalities used for fusion imaging in a comprehensive, standardized setting. The maximum acceptable error of registration in FI is debatable. While deviations of several millimeters may be acceptable for utilization during endovascular therapy of peripheral arteries, complex endovascular procedures, such as fenestrated or branched endovascular aneurysm repair (EVAR), and visceral artery revascularization, carry a higher risk of potentially fatal complications. Lalys and colleagues suggested a clinically acceptable deviation of 3 mm for an EVAR procedure based on the mean size of the renal artery ostium of 6 mm (Lalys et al. 2019), which has been adopted by other authors(Schulz et al. 2016; Schulz et al. 2019; Lalys et al. 2019). Schulz and colleagues compared both fusion techniques on CT data sets in EVAR procedures on 50 patients using the craniocaudal deviation to the lower renal artery ostium. Although both techniques’ insignificant deviation was reported (3D-3D: 3.6 ± 3.9 mm; 2D-3D: 4.6 ± 4.4 mm), an evident risk of over-stenting an ostium was observed in 12% of patients in the 2D-3D cohort. Additionally, in 88% of cases, a caudal deviation of the virtual 3D vessel model was observed, resulting in potential misregistration of the landing zone. In 46%, this deviation was more than 5 mm (Schulz et al. 2019). This is in concordance with our study’s findings, which showed mean deviations of up to 4.47 ± 0.92 mm for 2D-3D registration; however, at individual measurement points, the deviation was close to 6 mm. By contrast, registration errors for 3D-3D registration were markedly lower at 1.67 ± 0.24 mm in our study, while most means were either close to or below 1 mm. Further factors influencing the registration accuracy, such as different positions of the patient’s arms during the pre-interventional CT/MRI examinations and the interventional procedure, and respiratory movements could not be reproduced in our study. While the reported impact of these conditions in the literature is < 2 mm each (Doyle et al. 2017; Draney et al. 2005), these add up to a potential error of > 9 mm using 2D-3D registration based on our data. For 3D-3D registration, the sum of errors would not surpass 5 mm, except for two measurement points in the infrarenal aneurysm. Consequently, the use of 2D-3D registration, at least for the guidance of central body deployment cannot be recommended based on our data, whereas 3D-3D registration may offer the necessary accuracy under optimal conditions. Other characteristics that vary from patient to patient, such as the distance between the aneurysm and the renal or the supraaortic arteries, maybe the decisive factors here. Furthermore, interventions of the iliac arteries are a promising field since inaccuracies due to respiratory movements play a lesser role. The superiority of 3D-3D registration is underlined by the results regarding landmark placement. The cannulation of vessels is also a time-consuming and radiation-intensive work step, for which the fusion technique can assist. Since there is no risk of permanently occluding vessel ostia with stent grafts and the risk of vessel injury can be minimized by the experienced interventionalist through careful management of guidewires and catheters, lower registration accuracies might also be acceptable here.

In addition to saving on CM and improving patient safety, radiation hygiene is a major FI goal. This is where 3D-3D technology shows certain drawbacks. For example, radiation doses for 3D-3D FI of 45.7 ± 9.1 Gy*cm^2^ compared to 0.45 ± 0.26Gy*cm^2^ for 2D-3D registration were reported (Schulz et al. 2019). Similar observations were made by van den Berg et al. (Van de Berg 2013). From a practical perspective, 2D-3D registration may provide a sufficient basis for initial orientation and vessels’ cannulation. Considering that none of the above-mentioned in vivo studies recommend stent placement based on fusion imaging alone, a sequential approach may be employed. Initial 2D-3D fusion, cannulation of target vessels, targeted angiograms using small doses of CM, adaptation of the image registration, and subsequent stent-graft placement would be conceivable.

The results of our study confirm that image modalities have no relevant influence on the accuracy of fusion imaging. The spread shown in the Bland Altman analysis and the limits of agreement were each within the range reported for the two registration techniques. However, reasons for these deviations remain speculative. Other researchers have reported increased inaccuracies in the thorax compared to the abdomen, which could not be reproduced in our study and may be attributed to increased motion errors from breathing and cardiac motion (Schulz et al. 2016; Abi-Jaoudeh et al. 2012; Carrell et al. 2010; Fukuda et al. 2013). Stahlberg et al. described notable inaccuracies for quantitative measurements of > 20% in the peripheral arteries, which were attributed to a magnification effect due to variable distances between target structures and the X-ray tube (Stahlberg et al. 2018). A magnifying effect on anatomical target structures used to adjust the virtual 3D vascular model in 2D may explain possible deviations. We also observed an increasing deviation of the registration accuracy in oblique angulations of 30°–60° RAO/LAO that suggest a systematic, possibly software related error.

In perspective, models that allow for automated, dynamic adaptation of the 3D model to the actual anatomy may further reduce radiation doses and the use of CM. Numerical models based on biomechanical data or radiopaque markers can be used to predict the displacement of vascular structures (Dumenil et al. 2013; Guyot et al. 2013; Gindre et al. 2015). Alternatively, markers positioned externally on the patient can be used for motion correction (Koutouzi et al. 2016). The tracking of introduced devices to predict alterations in anatomy is also a promising approach.

This work’s limitations can primarily be attributed to the use of a phantom instead of patient data. The use of a phantom allowed for the performance of fusion-guided procedures several times under standardized conditions with low bias and without having to consider radiation exposure or complication risks; however, other aspects known to impair fusion imaging, such as patient positioning and different breathing positions, could not be reproduced artificially. As we used only a single vessel model, different vessel characteristics with a known influence on fusion accuracy, such as aneurysm morphology, were not assessed in this study. Vessel displacement by inserting stiff devices was not investigated as vessel compliance has high interindividual differences.

## Conclusion

This study demonstrated that CTA and MRA could be used equivalently for FI. In the realm of registration, the 3D-3D method proved more accurate than did the 2D-3D method. The error analysis of different registration techniques in this study allows the choice of the registration method to be better adapted to the requirements of endovascular image-guided procedures in the future.

## Data Availability

The data generated and analysed in this study is included in the manuscript.

## Abbreviations

FI: Fusion imaging
CM: Contrast medium
2D: 2-dimensional
3D: 3-dimensional
RAO: Right anterior oblique
LAO: Left anterior oblique
ICC: Intralcass correlation coefficient
